# Interactions between *ACYP2* genetic polymorphisms and environment factors with susceptibility to ischemic stroke in a Han Chinese Population

**DOI:** 10.18632/oncotarget.18430

**Published:** 2017-06-09

**Authors:** Qiong Cheng, Yong-Kun Li, Feng Lu, Lianhua Yin, Yin-Zhou Wang, Wen Wei, Qian Lin

**Affiliations:** ^1^ Department of Neurology, Fujian Provincial Hospital, Provincial Clinical Department of Fujian Medical University, Fuzhou, 350001, Fujian Province, China; ^2^ The Second People's Hospital Affiliated to Fujian University of Traditional Chinese Medicine, Fuzhou, 350003, Fujian Province, China; ^3^ Department of Rehabilitation of GanZhou Municipal Hospital, GanZhou, 341000, Jiangxi Province, China; ^4^ Department of Neurology, Fuzhou Second Hospital, Fuzhou, 350007, Fujian Province, China

**Keywords:** ischemic stroke, single nucleotide polymorphisms, interaction, smoking, alcohol drinking

## Abstract

**Aims:**

To investigate the association of several single nucleotide polymorphisms (SNPs) within *ACYP2* gene and additional gene- environment interaction with ischemic stroke (IS) risk in a Chinese population.

**Results:**

IS risk was significantly higher in carriers with the G allele of rs11896604 than those with CC genotype (CG or GG versus CC), adjusted OR (95%CI) =1.60 (1.18–2.20), and higher in carriers with the A allele of rs12615793 than those with GG genotype (GA or AA versus GG), adjusted OR (95%CI) = 1.66 (1.24–2.15). GMDR model shown a significant two-locus model (*p* = 0.0010) involving rs11896604 and alcohol drinking, and a significant two-locus model (*p* = 0.0010) involving rs12615793 and smoking. Current smokers with rs12615793- GA or AA genotype have the highest IS risk, compared to never- smokers with rs12615793-GG genotype, OR (95%CI) = 2.72 (1.64–3.86); current drinkers with rs11896604-CG or GG genotype have the highest IS risk, compared to never- drinkers with rs11896604-CC genotype, OR (95%CI) = 2.51 (1.70–3.40).

**Materials and Methods:**

A total of 1202 participants (660 males, 542 females) were selected, including 600 IS patients and 602 control participants. The mean age of all participants was 68.2 ± 15.8 years. Generalized multifactor dimensionality reduction (GMDR) was used to screen the best interaction combination. Logistic regression was performed to investigate the impact of 4 SNPs within *ACYP2* gene, additional gene-smoking or drinking interaction on IS risk.

**Conclusions:**

We found that the G allele of rs11896604 and the A allele of rs12615793 within *ACYP2* gene, rs12615793- smoking interaction, and rs11896604-alcohol drinking interaction were all associated with increased IS risk.

## INTRODUCTION

Stroke is caused by cell death owing to abnormal blood supply in brain with high mortality and disability [1, 2]. In China, the stroke prevalence and death rate was much higher than western countries [3, 4]. Among all subtypes of stroke, ischemic stroke (IS) have accounted for the majority of cases in China [5]. IS was a common type, accounts for 85% of stroke deaths and was effected by multiple factors, such as gene, hypertension, tobacco smoking and diabetes [6]. Previous studies have reported that the shorter telomere length (TL) was associated with risk of several diseases, including IS [7, 8].

The *ACYP2* gene which located on chromosome 2p16.2, encodes a small cytosolic acylphosphatase enzyme that catalyzes the hydrolysis of carboxyl-phosphate bonds [9]. Genome wide association study [10] have demonstrated that genetic polymorphisms in *ACYP2* are associated with telomere length, which has led to studies of the association between *ACYP2* and various diseases, including ischemic stroke [7, 8]. A recent paper has indicated a significant association between *ACYP2* and *TSPYL6* single nucleotide polymorphisms (SNPs) associated with coronary heart disease (CHD) [11]. To date, just one study has reported the association between *ACYP2* gene SNPs and IS risk in Chinese population. In addition, IS susceptibility was influenced by both genetic and environment factors, such as smoking and drinking [12]. But till now, no study focused on the synergistic effect between *ACYP2* gene and smoking or drinking on IS risk. So the aim of this study was to investigate the impact of several SNPs within *ACYP2* gene, and their additional gene-smoking or gene- alcohol drinking interaction on IS risk in this Chinese population.

## RESULTS

A total of 1202 subjects (660 males, 542 females) were selected, including 600 IS cases and 602 controls. The mean age was 68.2 ± 15.8 years for all studied participants. Table 1 shows the general characteristics for case and control group. There were no significant difference between cases and controls in mean age and distributions for males, hypertension and T2DM were not significantly different. The mean of BMI and the rates of smokers and alcohol drinkers were higher in cases than controls.

**Table 1:** General characteristics of 1202 study participants in case and control group

Variables	Case group (*n* = 600)	Control group (*n* = 602)	*p*-values
Age (year)	67.8 ± 15.5	68.5 ± 16.2	0.444
Males, N (%)	328 (54.7)	332 (55.5)	0.866
Smoking, N (%)	203 (33.8)	161 (26.7)	0.007
Alcohol consumption, N (%)	287 (47.8)	206 (34.2)	< 0.001
BMI (kg/m^2^)	24.8 ± 9.1	23.3 ± 9.0	0.004
Hypertension (%)	218 (36.3)	206 (34.2)	0.443
T2DM (%)	120 (20.0)	130 (21.6)	0.496

Note: Means ± standard deviation for age, WC, BMI; WC, waist circumference; BMI, body mass index.

All *P-* values for the Hardy–Weinberg equilibrium test were more than 0.05. The frequencies for the G allele of rs11896604 and the A allele of rs12615793 were significantly higher in IS patients than controls (30.9% *vs*20.3%, 29.0% *vs*19.5%). IS risk was significantly higher in carriers with the G allele of rs11896604 than those with CC genotype (CG or GG versus CC), adjusted OR (95%CI) = 1.60 (1.18–2.20), and higher in carriers with the A allele of rs12615793 than those with GG genotype (GA or AA versus GG), adjusted OR (95%CI) = 1.66 (1.24–2.15). However, rs843711 and rs11125529 were not associated with IS risk after covariates adjustment. (Table 2)

**Table 2:** Genotype and allele frequencies of 4 SNPs between case and control group

SNP	Genotypes and Alleles	Frequencies N (%)		OR (95%CI)^*^	*P*-values	*P*-values for HWE test in controls
		Control (*n* = 602)	Case(*n* =600)			
rs843711						
	Co-dominant					
	CC	352 (58.5)	313 (52.2)	1.00 (ref)		0.953
	CT	217 (36.0)	236 (39.3)	1.23 (0.80−1.81)	0.486	
	TT	33 (5.5)	51 (8.5)	1.47 (0.75−2.27)	0.521	
	Dominant					
	CC	352 (58.5)	313 (52.2)	1.00 (ref)		
	CT+TT	250 (41.5)	287 (47.8)	1.30 (0.78−1.90)	0.516	
	Allele, T (%)	283 (23.5)	338 (28.2)			
rs11896604						
	Co-dominant					
	CC	388 (64.4)	297 (49.5)	1.00 (ref)		0.183
	CG	184 (30.6)	235 (39.2)	1.35 (1.12−1.79)	< 0.001	
	GG	30 (5.0)	68 (11.3)	2.10 (1.44–3.04)	< 0.001	
	Dominant					
	CC	388 (64.4)	297 (49.5)	1.00 (ref)		
	CG+GG	214 (35.6)	303 (50.5)	1.60 (1.18−2.20)	< 0.001	
	Allele, G (%)	244 (20.3)	371 (30.9)			
rs12615793						
	Co-dominant					
	GG	394 (65.4)	308 (51.3)	1.00 (ref)		0.291
	GA	181 (30.1)	236 (39.3)	1.45 (1.20−1.83)	< 0.001	
	AA	27 (4.5)	56 (9.3)	2.10 (1.41−2.85)	< 0.001	
	Dominant					
	GG	394 (65.4)	308 (51.3)	1.00 (ref)		
	GA+AA	208 (34.6)	292 (48.7)	1.66 (1.24−2.15)	< 0.001	
	Allele, A (%)	235 (19.5)	348 (29.0)			
rs11125529						
	Co-dominant					
	CC	345 (57.3)	307 (51.2)	1.00 (ref)		0.220
	CA	214 (35.6)	231 (38.5)	1.19 (0.79−1.74)	0.607	
	AA	43 (7.1)	62 (10.3)	1.41 (0.73–2.14)	0.823	
	Dominant					
	CC	345 (57.3)	307 (51.2)	1.00 (ref)		
	CA+AA	257 (42.7)	293 (48.8)	1.24 (0.76−1.89)	0.753	
	Allele, A (%)	300 (24.9)	355 (29.6)			

^*^Adjusted for gender, age, smoking and alcohol status, BMI and WC. Bonferroni correction threshold: *p* < 0.00625.

GMDR model were used to screen the best interaction combinations for gene- smoking and gene- alcohol drinking interaction. Table 3 shown a significant interaction between rs11896604 and alcohol drinking (*p* = 0.0010), the cross-validation consistency and the testing accuracy of this two- locus model were 10/ 10 and 60.72% respectively. We also found a significant two-locus model (*p* = 0.0010) involving rs12615793 and smoking, the cross-validation consistency of this two- locus model was 10/ 10, and the testing accuracy was 60.11%. In the logistic regression stratified analysis for the significant models. We found that current smokers with rs12615793- GA or AA genotype have the highest IS risk, compared to never- smokers with rs12615793- GG genotype, OR (95% CI) = 2.72 (1.64–3.86), after covariates adjustment; current drinkers with rs11896604- CG or GG genotype have the highest IS risk, compared to never- drinkers with rs11896604- CC genotype, OR (95%CI) = 2.51 (1.70–3.40), after covariates adjustment. (Table 4)

**Table 3:** GMDR analysis on the best gene–environment interaction models

Locus no.	Best combination	Cross-validation consistency	Testing accuracy	*p*-values
Gene- alcohol drinking interactions^*^				
2	rs11896604 alcohol drinking	10/10	0.6072	0.0010
3	rs11896604 rs11896604 alcohol drinking	7/10	0.5399	0.1719
4	rs11896604 rs11896604 rs11125529 alcohol drinking	6/10	0.5399	0.3770
5	rs11896604 rs11896604 rs11125529 rs843711 alcohol drinking	6/10	0.4958	0.4258
Gene- smoking interactions^**^				
2	rs12615793 Smoking	10/10	0.6011	0.0010
3	rs12615793 rs11896604 Smoking	8/10	0.5399	0.0547
4	rs12615793 rs11896604 rs843711 Smoking	7/10	0.5399	0.1719
5	rs12615793 rs11896604 rs843711 rs11125529 Smoking	6/10	0.4958	0.4258

^*^Adjusted for gender, age, smoking and BMI

^**^Adjusted for gender, age, drinking and BMI.

**Table 4:** Stratified analysis for gene-environment interaction by using logistic regression

Variable 1	Variable 2	OR (95% CI)^*^	*P-*values
rs11896604	Alcohol drinking		
CC	Never	1.00	−
CG+GG	Never	1.31 (1.01−1.73)	0.038
CC	Current	1.40 (1.10−1.82)	0.002
CG+GG	Current	2.51 (1.70−3.40)	< 0.001
rs12615793	Smoking		
GG	Never	1.00	−
GA+AA	Never	1.43 (1.04−1.87)	0.022
GG	Current	1.56 (1.17−2.02)	< 0.001
GA+AA	Current	2.72 (1.64−3.86)	< 0.001

^*^Adjusted for gender, age, drinking and BMI.

## DISCUSSION

In this study, we found that both the G allele of rs11896604 and the A allele of rs12615793 were significantly related with increased IS risk. However, rs843711 and rs11125529 were not associated with IS risk after covariates adjustment. To date, just one study [13] have reported the association between SNPs within *ACYP2* gene and IS risk. The authors suggested a significant association of *ACYP2* and *TSPYL6* polymorphisms with IS risk. However, the sample size of this study was relatively small, to validate the relationship between *ACYP2* and *TSPYL6* SNPs and IS susceptibility. Codd et al. [10] successfully confirmed that ACYP2 gene was associated with TL, they identified the association of rs11125529 in *ACYP2* genes was associated with shorter TL in the European population. Previously, several studies have reported the association between *ACYP2* gene and some other diseases, such as lung cancer [14], colorectal cancer (CRC) [15], high altitude pulmonary edema (HAPE) [16] and coronary heart disease (CHD) [17]. Chen et al. [14] identified that several SNPs within *ACYP2*, including rs1682111, rs11896604 and rs843720, were associated with lung cancer in the Chinese Han population, they suggested *ACYP2* may be a useful marker that informs clinical decisions, and may shed light on new candidate genes and new ideas about the mechanism governing the occurrence of lung cancer. Liu et al. [15] indicate that SNPs in the *ACYP2* gene are associated with CRC in a Chinese Han population, and these SNPs may serve as a prognostic biomarkers for CRC in the Chinese population. Ding et al. [16] found that rs11125529 was associated with an age-related disease- CHD. A GWAS study conducted by Aouizerat et al. [11] indicated that *ACYP2-* rs1559040 was associated with sudden cardiac arrest in CAD patients. Besides, another two studies [17, 18] suggested a significant association between rs1872328 of *ACYP2* gene and susceptibility to cisplatin-induced hearing loss.

IS susceptibility was influenced by both genetic and environment factors, and previously several environmental factors associated with IS were reported, and in these risk factors, cigarette smoking and alcohol drinking have been suggested to play a crucial role in increasing the risk of IS risk. In current study, the rate of smoking and alcohol drinking were higher in IS cases than that in controls, so in this study we also investigated gene- smoking or drinking interaction on IS risk. We found a significant interaction involving rs12615793 and smoking, rs11896604 and alcohol drinking. Current smokers with rs12615793-GA+AA genotype have the highest IS risk, compared to never- smokers with rs12615793- GG genotype; current drinkers with rs11896604- CG+GG genotype have the highest IS risk, compared to never- drinkers with rs11896604-CC genotype. The results of this study suggest that the risk of IS may be modified by some lifestyle factors, such as smoking and alcohol drinking. These genetic variants (rs12615793 and rs11896604) could influence susceptibility to IS risk, interact with smoking or alcohol drinking.

There several limitations in our study. Firstly, more SNPs within *ACYP2* gene should been included in the analysis, not only for just 4 SNPs. Secondly, some others environmental risk factors should be investigated in the gene-environment interaction analysis. Thirdly, haplotype analysis should be performed in the future studies.

In conclusion, we found that the G allele of rs11896604 and the A allele of rs12615793 within *ACYP2* gene, interaction between rs12615793 and smoking, rs11896604 and alcohol drinking were all associated with increased IS risk.

## MATERIALS AND METHODS

### Subjects

All study participants were recruited between Jun 2012 and March 2016 from Fujian Provincial Hospital. All patients were diagnosed with computed tomography (CT) and/or magnetic resonance imaging (MRI) scan confirming the presence of infarction. Patients were excluded from the study if stroke was determined to have resulted from trauma or any invasive hemorrhage. The controls were randomly selected from a population, who received physical examination in our hospital and 1:1 matched to cases on the basis of age (± 3 years) and sex. Subjects with diabetes, hypertension and others related risks were not excluded from the controls. Both the IS cases and controls were unrelated Han Chinese population. All demographic and related clinical data including residential region, age, ethnicity, and education status were collected through a face-to-face questionnaire and a review of medical records. Body weight and height measured. Current cigarette smokers were those who self-reported smoking cigarettes at least once a day for 1 year or more. Current alcohol consumption was expressed as the sum of milliliters of alcohol per week from wine, beer, and spirits. Blood samples were collected from each participant in the morning after at least 8 hours of fasting. Informed consent was obtained from all participants.

### Genomic DNA extraction and genotyping

The SNPs were selected based on the NCBI database (http://www.ncbi.nlm.nih.gov/projects/SNP). Taking into account the limited human resources and financial resources, just 4 SNPs within *ACYP2* gene were selected for genotyping, including: rs11896604, rs11896604, rs11125529 and rs843711. Genomic DNA from participants was extracted from EDTA-treated whole blood, using the DNA Blood Mini Kit (Qiagen, Hilden, Germany) according to the manufacturer's instructions. Genotyping was performed using a Sequenom MassARRAY RS1000 (Sequenom, Inc.) according to the manufacturer's protocol [19]. The SequenomTyper 4.0 Software™ (Sequenom, Inc.) was used to manage and analyze the data [20]. The nucleotide sequence of primers and description for the 6 SNPs were shown in Table 5.

**Table 5:** Description and primer sequence for 4 SNPs used for PCR analysis

SNP ID	Chromosome	Functional Consequence	Major/ minor alleles	Primer sequences
rs843711	2:54251980	Intron variant	C/ T	1st-PCRP: ACGTTGGATGGACAAAGGACCTTACAACTC 2nd-PCRP: ACGTTGGATGTGCCTTGTGGGA ATTAGAGC UEP_SEQ: gggaTCAGGGAACCAGTGCAAA
**rs11896604**	2:54251980	Intron variant	C/ G	1st-PCRP: ACGTTGGATGAAGTCAGAATAGTGCTTAC 2nd-PCRP: ACGTTGGATGTGTCTCTGACCTAGCATGTA UEP_SEQ: GTTAAGCTTGCAAGGAG
**rs12615793**	2:54248777	Intron variant	G/ A	1st-PCRP: ACGTTGGATGTTTGAGCTTAGTTGTTTAC 2nd-PCRP: ACGTTGGATGATCTTGGCCCTTGAAGAA UEP_SEQ: AAATTGAGTGACAAATATAAACTAC
rs11125529	2:54248729	Intron variant	C/ A	1st-PCRP: ACGTTGGATGGAGCTTAGTTGTTTACAGATG 2nd-PCRP: ACGTTGGATGCCGAAGAAAAGAAGATGAC UEP_SEQ: AGAAAAGAAGATGACTAAAACAT

### Statistical analysis

All statistical analyses were performed using the SPSS 22.0 software package (SPSS Inc, Chicago) for Windows 7. Categorical variables were presented as absolute values and percentages, and continuous variables were expressed as means ± standard deviation (SD). Student's *t* test was used to compare continuous variables, while Chi-square test was used to compare categorical variables between cases and controls. Hardy-Weinberg equilibrium (HWE) examination was used by SNPstats (http://bioinfo.iconcologia.net/SNPstats). Generalized multifactor dimensionality reduction (GMDR) model was used to analyze the gene- gene and gene- environment interaction, some parameters including cross-validation consistency, the testing balanced accuracy and the sign test were calculated, a sign test or a permutation test (providing empirical *p*-values) for prediction accuracy can be used to measure the significance of an identified model. Logistic regression was performed to investigate association between 4 SNPs within *ACYP2* gene and IS risk, and used for stratified analysis on significant interaction combination obtained from GMDR. All reported *p*-values were two-tailed, and to correct for multiple testing we defined a Bonferroni corrected- threshold in different tables.
